# Therapeutic advances in type 1 myotonic dystrophy complicated with type 2 diabetes mellitus

**DOI:** 10.3389/fneur.2025.1640563

**Published:** 2025-09-11

**Authors:** Lin Luo, Changsen Zhu, Shaona Yang, Yuan Sun, Muyang Rong, Tianrong Li

**Affiliations:** ^1^Department of Geriatrics, Kunming University of Science and Technology, The First People's Hospital of Yunnan Province, Kunming, China; ^2^Department of Otolaryngology, Kunming University of Science and Technology, The First People's Hospital of Yunnan Province, Kunming, China; ^3^Department of Clinical Laboratory, Kunming University of Science and Technology, The First People's Hospital of Yunnan Province, Kunming, China; ^4^Department of Obstetrics and Gynecology, Kunming University of Science and Technology, The First People's Hospital of Yunnan Province, Kunming, China

**Keywords:** type 1 myotonic dystrophy, type 2 diabetes, insulin resistance, pathogenesis, diagnosis and treatment

## Abstract

Myotonic Dystrophy (DM) is a hereditary muscle disorder characterized by progressive muscle weakness, myotonia, and multi-system dysfunction. Based on clinical and genetic features, DM can be classified into Type 1 (Type 1 Myotonic Dystrophy, DM1) and Type 2 (Type 2 Myotonic Dystrophy, DM2), with DM1 being the most common subtype in adulthood. Diabetes, a metabolic disease, is defined by persistent hyperglycemia, typically resulting from insufficient insulin secretion or impaired insulin action. Among the various forms of diabetes, Type 2 Diabetes (T2DM) has the highest prevalence, accounting for approximately 90% of all cases. Research has shown that individuals with Myotonic Dystrophy Type 1 (DM1) often experience comorbid Type 2 Diabetes (T2DM), a phenomenon that not only significantly increases the clinical burden but is also closely associated with poor prognosis, severely impacting patients’ quality of life. This review provides a comprehensive analysis of the latest research on insulin resistance in DM1 patients, shedding light on the underlying mechanisms of DM1-related T2DM. Additionally, it explores the common comorbidities shared by DM1 and T2DM, including those affecting the muscular, respiratory, cardiovascular, endocrine, and nervous systems, as well as cancer and depression. Finally, this article summarizes the most recent therapeutic strategies for managing DM1 with T2DM, focusing on glucose-lowering medications combined with emerging targeted therapies that address the core pathophysiology of DM1, showing promising preclinical outcomes. This review aims to provide a theoretical foundation for future research and clinical practice in the management of DM1 complicated by T2DM.

## 1 Introduction

The global overall prevalence of Type 1 Myotonic Dystrophy (DM1) is estimated to be 1 in 20,000 (1). Its pathogenesis is closely associated with abnormalities in the Dystrophia Myotonica Protein Kinase (DMPK) gene located on chromosome 19q13.3. The disease is caused by the expansion of the CTG trinucleotide repeat sequence in the non-coding region of the gene, with pathogenic expansions typically exceeding 50 repeats (2). Existing studies have shown that the degree of CTG repeat expansion is significantly correlated with clinical manifestations: the greater the repeat length, the more severe the clinical symptoms, the earlier the onset age, and the worse the prognosis (3, 4). The disease is primarily characterized by progressive skeletal muscle weakness, myotonia, and muscle atrophy, while also affecting multiple organ systems (5). According to the 10th edition of the Global Diabetes Epidemiology Report published by the International Diabetes Federation (IDF) in 2021 (6), the total number of adults aged 20–79 years with diabetes worldwide has reached 537 million, with an overall prevalence of 10.5%. In China, the prevalence of Type 2 diabetes among adults is 11.6%, with approximately 141 million affected individuals. Epidemiological projection models suggest that, without effective interventions, the global number of diabetes patients may rise to 783 million by 2045. Type 2 diabetes, also known as non-insulin-dependent diabetes or adult-onset diabetes, is primarily characterized by elevated blood glucose levels, insulin resistance, and relative insulin deficiency (7), and it accounts for the majority of all diabetes cases.

In a large cohort study involving 913 patients with Type 1 Myotonic Dystrophy (DM1) and 12,318 matched controls without DM1 (8), the prevalence of Type 2 diabetes in DM1 patients (8%) was significantly higher than in the matched controls (3%), approximately three times greater. This finding is consistent with data from a large study in France involving 1,409 DM1 patients (9), further indicating a significantly elevated risk of Type 2 diabetes in individuals with DM1. Notably, a retrospective study (10) observed two cases of congenital Type 1 Myotonic Dystrophy (DM1) in which diabetes was diagnosed during adolescence, suggesting that individuals with DM1 may experience an earlier onset of diabetes. Current research on the coexistence of Type 1 Myotonic Dystrophy and Type 2 diabetes is still in the early exploratory stage, particularly regarding personalized treatment strategies and the development of potential targeted therapies, which require further evidence from clinical studies. Therefore, this review explores the advances in the diagnosis and management of DM1 complicated by Type 2 diabetes from multiple perspectives, with the aim of providing a theoretical foundation for clinical practice.

## 2 Pathogenic mechanisms underlying type 1 myotonic dystrophy complicated with type 2 diabetes

Multiple studies have shown that the pathogenic mechanism of Type 1 Myotonic Dystrophy (DM1) primarily involves RNA-mediated toxicity. The transcription of expanded repeat sequences generates mutant RNAs, which alter the splicing of various target transcripts, leading to the complex clinical manifestations of DM1. This mechanism affects nearly all cells and organs in the body (11, 12). Based on clinical presentation and age of onset, Type 1 Myotonic Dystrophy (DM1) can be classified into three main subtypes: congenital, classic, and mild. In congenital DM1, patients may exhibit abnormal pregnancy signs such as polyhydramnios and decreased fetal movement during the prenatal stage. At birth, they often present with hypotonia, severe generalized weakness, respiratory insufficiency, and a high risk of early mortality, with intellectual disability being common. Classic DM1 is characterized by typical muscle damage and multi-system dysfunction, with adults potentially experiencing physical disability and shortened lifespan. Mild DM1 typically manifests with cataracts, diabetes, and mild muscle stiffness (persistent muscle contractions), but patients have a normal life expectancy. Genetic testing is the gold standard for diagnosing DM1, while amniocentesis, muscle biopsy, serum creatine kinase levels, and electromyography (EMG) can serve as supplementary diagnostic methods (10, 13–15).

The risk of developing diabetes is increased 2–4 times in patients with Type 1 Myotonic Dystrophy (DM1) (16). Several studies (17, 18) have assessed insulin resistance using the Homeostatic Model Assessment of Insulin Resistance (HOMA-IR) and found that DM1 patients exhibit elevated fasting plasma insulin levels (i.e., hyperinsulinemia) and significantly higher HOMA-IR scores compared to healthy controls. These findings suggest that insulin resistance is the primary pathophysiological mechanism underlying the impaired glucose metabolism observed in DM1. Insulin resistance refers to a pathological state in which the sensitivity and responsiveness of target tissues—primarily skeletal muscle, liver, and adipose tissue—are reduced, leading to a decreased efficiency of insulin-mediated glucose uptake and utilization (19, 20). In patients with Type 1 Myotonic Dystrophy (DM1), insulin resistance not only increases the risk of developing Type 2 diabetes but may also be associated with core pathological processes such as muscle atrophy. Therefore, a deeper understanding of its underlying mechanisms is critical for developing effective intervention strategies (21–23). Current research primarily explores the complex mechanisms of insulin resistance in DM1 from multiple perspectives, including RNA toxicity-induced alternative splicing abnormalities, post-receptor signaling pathway dysfunction, loss of DMPK protein function, and disruptions in inflammation and metabolism.

### 2.1 RNA toxicity and selective splicing abnormalities of the insulin receptor (INSR) are core mechanisms involved

The insulin receptor (IR) gene generates two functional isoforms, IR-A and IR-B, through alternative splicing. While both isoforms are expressed in most cells, the IR-B isoform predominates in insulin-sensitive tissues such as adipose tissue, liver, and skeletal muscle (24, 25). In Type 1 Myotonic Dystrophy (DM1), the mutated DMPK mRNA is retained in the nucleus, where the expanded 3′ trinucleotide repeat forms nuclear hairpin structures. These structures sequester various RNA-binding proteins, particularly Muscleblind-like protein 1 (MBNL1), which plays a key role in regulating mRNA splicing (26, 27). The sequestration of MBNL1 disrupts its typical function in transcripts associated with mitochondrial structure and dynamics, such as OPA1 (optic atrophy 1) and Mitofusin-2 (MFN2), both of which are crucial for mitochondrial fusion (28, 29). This splicing alteration results in increased mitochondrial fission, leading to fragmented, swollen, and disorganized cristae structures that accumulate around the nucleus (27, 30). Moreover, these structural abnormalities are closely associated with impaired mitochondrial function, including reduced ATP synthesis, increased oxidative stress, and compromised mitochondrial activity, ultimately triggering cellular dysfunction (31, 32). Meanwhile, DMPK mRNA containing the expanded CTG repeat sequence accumulates in the nucleus, which may also lead to dysfunction of other regulatory factors, such as CUGBP-Elav-like family member 1 (CUGBP1 or CELF1). However, unlike MBNL1, CUGBP1 is not sequestered by the expanded repeats, but instead undergoes excessive phosphorylation mediated by various protein kinases, such as Protein Kinase C (PKC), Cyclin D3 (CCND3), and Cyclin-dependent Kinase 4 (CDK4) (33, 34). These kinases alter CUGBP1 by affecting its stability, localization, and activity. The hyperphosphorylation of CUGBP1 enhances its activity, leading to alternative splicing, and ultimately disrupting mRNA translation and transcript decay (35). However, CUGBP1 itself is not sequestered by the toxic RNA repeat sequence (36). The result is a loss of function and abnormal processing of many gene products, including muscle-specific chloride channels and the insulin receptor.

In skeletal muscles of patients with Type 1 Myotonic Dystrophy (DM1), the splicing pattern of the insulin receptor (INSR) gene undergoes a pathological shift, resulting in a significant increase in the expression of the IR-A isoform, which has lower metabolic signaling capability, while the IR-B isoform is reduced (37–39). Studies have shown that in skeletal muscle tissues from DM1 patients and cultured myocytes, the IR-A isoform predominates, and these cells exhibit a significantly reduced metabolic response to insulin compared to normal controls (18). Furthermore, the study found that overexpression of Cytidine Uridine Guanosine Binding Protein (CUG-BP) in normal cells induced the same shift in IR splicing patterns, suggesting that dysfunction of splicing regulators is a key factor contributing to insulin resistance in DM1. Subsequent studies have further confirmed that a relative increase in IR-A expression is observed in both type I and type II muscle fibers of DM1 and DM2 patients, indicating that this splicing abnormality is widespread and not specific to any particular muscle fiber type (37). This splicing alteration directly impairs skeletal muscle sensitivity to insulin, serving as an important molecular basis for insulin resistance in DM1 patients (40, 41).

### 2.2 Disruption of post-insulin receptor signaling pathways exacerbates insulin resistance

In addition to the splicing abnormalities at the receptor level, increasing evidence suggests that defects in the post-insulin receptor signaling pathways also play a crucial role in insulin resistance in DM1. Even when insulin binds to its receptor, the effective transmission of downstream signals may be impaired. A 2017 study, which analyzed skeletal muscle biopsies and myotube cultures from DM1 and DM2 patients, found alterations in the basal phosphorylation levels of key signaling molecules, including Akt/PKB, p70S6K, GSK3β, and ERK1/2. Furthermore, under insulin stimulation, the ability to activate proteins and facilitate glucose uptake was significantly reduced compared to controls. This suggests that post-receptor signaling abnormalities, independent of INSR splicing defects, are also a significant contributor to insulin resistance (42). Another study also found that the expression of the insulin receptor in type I fibers of skeletal muscles from DM1 and DM2 patients is reduced, leading to impaired activation of the insulin signaling pathway. This signaling defect further results in decreased mTOR activation, while promoting the increased expression of genes associated with muscle atrophy, such as MuRF1 and Atrogin-1/MAFbx. This links insulin resistance with muscle wasting in DM1 (22). These findings suggest that insulin resistance in DM1 is a multifaceted pathological process, involving multiple steps from receptor dysfunction to downstream effector molecules.

### 2.3 Potential effects of DMPK protein kinase dysfunction

Although RNA toxicity is widely recognized as a primary mechanism, the contribution of DMPK protein dysfunction to insulin resistance should not be overlooked. A study using a DMPK gene knockout (dmpk−/−) mouse model revealed that dmpk−/− mice exhibited impaired insulin signaling, glucose intolerance, and reduced insulin-dependent GLUT4 transport in muscle tissue, a phenomenon not observed in fat and liver tissues, which do not express DMPK. The study further demonstrated that DMPK is essential for the proper intracellular transport of both the insulin receptor and insulin-like growth factor 1 receptor (IGF-1R). These findings suggest that reduced expression of DMPK protein may directly impair insulin action in muscle, thereby promoting the development of insulin resistance (43). Another study conducted in DMPK gene knockout mice fed a high-fat diet found that, compared to wild-type mice, these knockout mice exhibited more pronounced weight gain, adipocyte hypertrophy, and exacerbated systemic insulin resistance. These findings suggest that DMPK deficiency may serve as a genetic risk factor, increasing susceptibility to obesity and insulin resistance induced by a high-fat diet (44).

### 2.4 The interplay between inflammation, metabolic dysregulation, and insulin resistance

It is noteworthy that patients with DM1 exhibit chronic low-grade inflammation and complex metabolic dysregulation, both of which may exacerbate insulin resistance.

1. The Role of Tumor Necrosis Factor-*α* (TNF-α): Studies have confirmed that the dysfunction of the RNA-binding protein CUGBP1 is responsible for the increased stability of TNF-α mRNA. TNF-α is a potent pro-inflammatory cytokine, and its overexpression in muscle tissue may exacerbate the muscle atrophy and insulin resistance characteristic of DM1 (23). Another study also observed elevated tumor necrosis factor system activity in DM1 patients, which was associated with abnormal protein degradation (45).
2. Abnormal Adiponectin Levels: Research has shown that, compared to healthy controls, DM1 patients exhibit significantly lower total serum adiponectin concentrations as well as reduced levels of its biologically active high-molecular-weight oligomers. Adiponectin is a hormone secreted by adipocytes that plays a role in improving insulin sensitivity. The decrease in adiponectin levels is closely associated with increased insulin resistance indices and elevated triglyceride levels in DM1 patients, suggesting that the reduction in adiponectin and its oligomers may contribute to the onset and progression of metabolic complications in DM1 (46).
3. Imbalance of Leptin and Testosterone Levels: Studies have shown that serum leptin levels in DM1 patients are significantly higher than those in healthy controls, irrespective of body fat content. At the same time, testosterone levels in male patients are lower. Moreover, hyperleptinemia in DM1 patients is significantly associated with hypogonadism. Genetic defects may directly or indirectly lead to elevated leptin levels, while increased cytokines may promote insulin resistance and other endocrine disturbances (47).
4. Non-Alcoholic Fatty Liver Disease (NAFLD): A prospective study involving 36 DM1 patients found that NAFLD is highly prevalent in DM1 patients, with an incidence rate of 44%. The condition is closely associated with insulin resistance (as indicated by significantly elevated HOMA-IR scores), abdominal obesity, and hypertriglyceridemia, which are characteristic features of metabolic syndrome. These findings further reinforce the central role of peripheral insulin resistance in the multi-organ complications of DM1 (48).

### 2.5 Compensatory and decompensatory insulin secretion in DM1 patients

**Figure 1:**
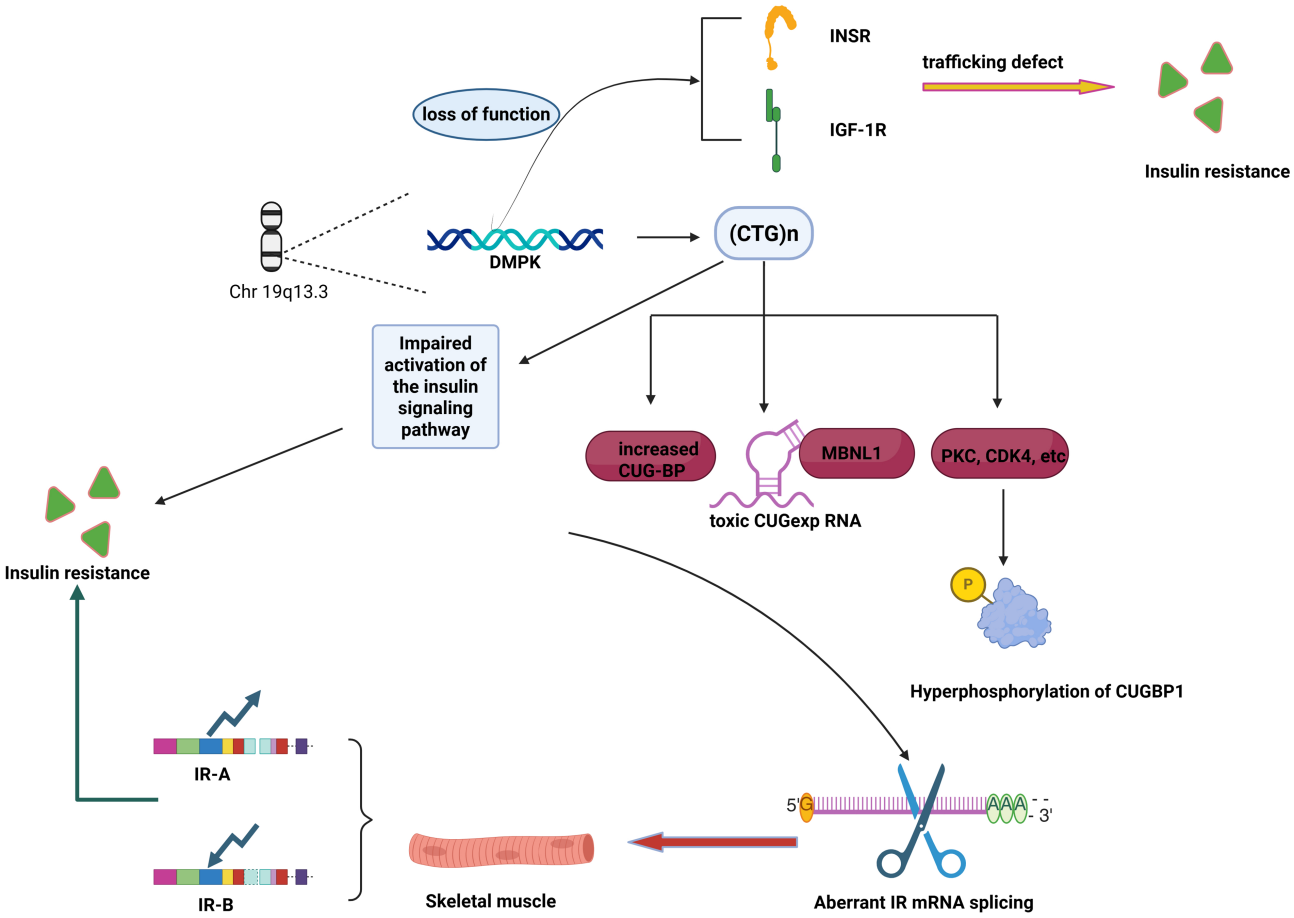
The main mechanism underlying insulin resistance in DM1 is the RNA toxicity caused by DM1 gene mutations, which leads to selective splicing abnormalities of the insulin receptor. In addition, impairments in post-insulin receptor signaling pathways exacerbate insulin resistance. Furthermore, the loss of function of DMPK protein kinase is also a significant contributing factor. Figure created in BioRender. Luo, L. (2025) https://BioRender.com/ z3tmhey.

Unlike typical type 2 diabetes, DM1 patients often exhibit a unique insulin secretion pattern during the early stages of impaired glucose tolerance. A cross-sectional study involving 95 DM1 patients found that even with low fasting blood glucose (FBS) levels (<80 mg/dL), DM1 patients still had higher HOMA-IR scores compared to the control group, indicating the presence of insulin resistance. During this stage, the insulin secretion index compensatorily increases, resulting in a state of hyperinsulinemia (17). Another study observed that, during oral glucose loading, young non-diabetic DM1 patients exhibited an enhanced early-phase insulin secretion response (49). However, as the disease progresses, this compensatory ability may gradually decline. Some studies have pointed out that insulin secretion in DM1 patients is abnormal, characterized by significantly elevated pre-fasting insulin levels and the insulin-to-glucose ratio, which is considered a marker of *β*-cell dysfunction. This dysfunction may play a more significant role than insulin resistance itself in the eventual progression to diabetes in these patients (49) (see Figure 1).

## 3 Common complications

Although type 1 myotonic dystrophy is characterized by muscle stiffness and RNA toxicity, and type 2 diabetes is primarily driven by insulin resistance, the two conditions have distinct etiologies. However, there is a significant overlap in terms of metabolic dysregulation and multi-system involvement. Both conditions can lead to similar complications affecting various systems, including the musculoskeletal, endocrine, cardiovascular, respiratory, and nervous systems.

### 3.1 Muscular system

The characteristic myopathic manifestations in patients with type 1 myotonic dystrophy include muscle weakness, muscle atrophy, and muscle stiffness. A study by Bouchard et al. (50) analyzed skeletal muscle involvement in 204 type 1 myotonic dystrophy patients and found that muscle strength decline was a universal phenomenon, strongly correlated with disease duration and the number of CTG repeats. A 2000 study (51) examining individual muscle fibers in type 1 myotonic dystrophy patients revealed that the specific strength of type I fibers was lower in all subjects compared to healthy controls, with symptomatic patients exhibiting lower type I fiber strength than asymptomatic patients. A 2006 study (52) also found that in patients with congenital type 1 myotonic dystrophy, one patient exhibited significant involvement of the tibialis anterior muscle, predominantly affected by type I fibers, resembling the muscle fiber distribution seen in older patients with congenital or classic type 1 myotonic dystrophy. These findings support the reduction and functional impairment of type I muscle fibers in type 1 myotonic dystrophy. Similarly, patients with type 2 diabetes often experience a decline in muscle strength and function. Filgueiras et al. (53) found that type 2 diabetes is associated with reduced grip strength and diminished knee extensor muscle strength. Additionally, type 2 diabetes patients showed an increased proportion of type II muscle fibers and a decreased proportion of type I fibers. These changes in muscle fiber composition may impact the patients’ muscle function and metabolic characteristics.

Volpato et al. (54) included 835 participants aged 65 years and older and found that diabetic participants had slower walking speeds in the 4-meter walk test, with a 24.3 and 15.1% difference in walking speeds for the 4-meter and 400-meter tests, respectively, when compared to non-diabetic participants. This suggests that gait and walking ability may be impaired in type 2 diabetes patients. In patients with type 1 myotonic dystrophy, muscle symptoms typically present with preferential involvement of distal limbs and facial muscles (55), indicating that gait and walking ability may also be compromised in these patients. Compared to individuals with normal blood glucose levels, patients with type 2 diabetes are at significantly higher risk of sarcopenia (56). Another study further confirmed that the accumulation of pre-mRNA processing factors in the muscle cell nuclei of both type 1 myotonic dystrophy and type 2 diabetes mellitus (DM2) patients resembles the nuclear changes observed in sarcopenia (57). These findings suggest that type 1 myotonic dystrophy and sarcopenia may share common cellular mechanisms that lead to skeletal muscle atrophy.

Therefore, when managing patients with type 1 myotonic dystrophy and type 2 diabetes, objective muscle function assessment tools should be employed to more accurately evaluate the patient’s muscular symptoms and functional status. Additionally, the potential impact of sarcopenia should be considered, providing a basis for improving disease outcomes and implementing personalized treatment strategies.

### 3.2 Endocrine system

Both type 1 myotonic dystrophy and type 2 diabetes are significantly associated with thyroid dysfunction, which may present with either hypothyroidism or hyperthyroidism (58, 59). Patients with type 1 myotonic dystrophy are at an increased risk of thyroid abnormalities and structural changes, with a higher likelihood of developing both benign and malignant thyroid nodules compared to the general population (60). Similarly, the prevalence of multinodular goiter is higher in patients with type 2 diabetes than in the general population (61). Interestingly, there is a potential overlapping pathological mechanism between thyroid dysfunction and type 2 diabetes, with thyroid hormone abnormalities potentially influencing the development of type 2 diabetes (62). Thyroid dysfunction may impair glucose tolerance and exacerbate metabolic disturbances in diabetic patients (61).

Multiple clinical studies have demonstrated that type 1 myotonic dystrophy (DM1) and type 2 diabetes are both associated with complex interactions involving various endocrine disorders. Patients with DM1 frequently present with multiple endocrine abnormalities, including hypergonadotropic hypogonadism, autoimmune thyroid disease, hyperinsulinemia, and disturbances in calcium–phosphate metabolism (63). A clinical study analyzing endocrine function in 97 DM1 patients confirmed that this population exhibits a higher incidence of parathyroid dysfunction and calcium–phosphate metabolic disorders (64). Data indicate that 40.8% of DM1 patients have gonadal dysfunction, often accompanied by impaired fertility, with male patients being particularly affected; adult primary testicular failure represents the most common endocrine abnormality in DM1 (62, 65). In patients with diabetes, the function of the entire endocrine system—including the hypothalamus, pituitary, adrenal glands, thyroid, parathyroid, vitamin D system, gonads, and adipose tissue—is compromised (66). Diabetes can affect multiple endocrine organs, particularly under conditions of poor glycemic control, with the most common consequences including growth retardation in children and adolescents and hypothalamic–pituitary–gonadal axis dysfunction in women (67).

### 3.3 Respiratory system

Troshina et al. (63) conducted a follow-up study over an average of 9.02 years involving 80 patients with type 1 myotonic dystrophy. The results indicated a slow but persistent decline in pulmonary function in these patients, with an annual change of: forced vital capacity (FVC) reduced by 0.034 ± 0.06 L (−0.72 ± 1.7% of predicted value) and forced expiratory volume in one second (FEV1) decreased by 0.043 ± 0.05 L (−1.07 ± 1.7% of predicted value). In a corresponding study, Anandhalakshmi et al. (68) compared pulmonary function in 30 patients with type 2 diabetes and 30 healthy controls. Data analysis revealed that pulmonary function parameters in diabetic patients, including forced vital capacity (FVC), forced expiratory volume in one second (FEV1), and peak expiratory flow rate (PEFR), were significantly lower than those in the control group. Another study (69) included 286 patients and, after multivariate analysis, found that the mean predicted percentage values of FEV1 and FVC in diabetic patients were significantly lower than those in non-diabetic patients. These findings suggest that the decline in pulmonary function may be a common respiratory complication in patients with type 1 myotonic dystrophy and type 2 diabetes. Diabetes may lead to reduced respiratory muscle strength, and neurodegenerative changes in the phrenic nerve can cause diaphragm paralysis, thereby impairing ventilatory function (70). A case report described a type 2 diabetes patient who developed neuropathy affecting the phrenic nerve, ultimately resulting in fatal respiratory failure (71). Although this condition is rare, it highlights the importance for clinicians to be vigilant for potential respiratory muscle dysfunction in patients with type 2 diabetes.

Patients with type 1 myotonic dystrophy commonly experience sleep-disordered breathing. A study found that 55% of type 1 myotonic dystrophy patients suffer from obstructive sleep apnea, with 12.5% exhibiting periodic breathing and 10% showing sleep hypoventilation (72, 73). In type 2 diabetes patients, sleep quality is significantly impaired, with approximately half of diabetic patients experiencing respiratory failure during sleep. This is particularly pronounced in those with autonomic neuropathy, where oxygen saturation decreases more severely and for prolonged durations (74).

The mechanisms underlying the impact of DM1 on the respiratory system are complex, involving multiple factors such as impaired central respiratory drive, peripheral respiratory muscle weakness, and dysfunction of the upper airway muscles (4). Respiratory muscle weakness is the core pathological basis for respiratory dysfunction in DM1. Numerous studies have confirmed that weakness of the respiratory muscles (including both inspiratory and expiratory muscles) is the primary cause of respiratory dysfunction in DM1 patients, directly leading to restrictive ventilatory impairment (75). Studies have shown (76, 77) that, compared to healthy controls, both maximal inspiratory pressure (MIP) and maximal expiratory pressure (MEP) are significantly reduced in DM1 patients. Ultrasound of the diaphragm reveals a significant decrease in the diaphragm thickening ratio (DTR), indicating impaired diaphragm function. The study also found that MIP and DTR are significantly correlated with the Muscle Impairment Rating Scale (MIRS), suggesting that the severity of respiratory muscle weakness aligns with the extent of systemic skeletal muscle involvement. Maximal voluntary ventilation (MVV) may serve as a sensitive indicator for screening ventilatory dysfunction in DM1 patients. In those with hypercapnia, the reduction in MVV predicted values is more pronounced than that of FVC (78).

In addition to peripheral respiratory muscle weakness, abnormalities in central respiratory drive regulation are also considered important mechanisms contributing to alveolar hypoventilation and hypercapnia in DM1 patients (75). DM1 patients exhibit a generally reduced ventilatory response to CO2 (0.85 ± 0.67 L/min/mmHg), and this reduction is not correlated with the severity of pulmonary dysfunction or the extent of respiratory muscle weakness. Based on this, researchers have proposed that DM1 patients may experience a central CO2 chemosensitivity disorder independent of peripheral factors (79, 80). However, while it is widely accepted that central drive plays a significant role in alveolar hypoventilation, the exact mechanisms remain unclear, and the current literature has yet to elucidate the underlying cause.

### 3.4 Cardiovascular system

Both type 1 myotonic dystrophy (DM1) and type 2 diabetes mellitus (T2DM) are associated with various cardiovascular complications. Although the specific overlapping comorbidities between the two conditions are not highly pronounced, cardiovascular disease remains one of the leading causes of mortality in both DM1 and T2DM patients. In DM1 patients, common cardiac abnormalities include conduction defects (e.g., first-degree atrioventricular block), arrhythmias, and, less frequently, heart failure, ischemic heart disease, and mitral valve prolapse (81). Cardiac conduction defects of varying severity are quite common. A retrospective study by Wahbi et al., based on data from 1,388 DM1 patients, identified age, a family history of sudden death, and left bundle branch block as independent predictors of sudden cardiac death (82). Cardiovascular complications commonly seen in type 2 diabetes mellitus (T2DM) patients include atherosclerotic cardiovascular disease (ASCVD) and heart failure (HF), which represent the primary fatal endpoints in this population. Notably, there is a significant difference in risk across age groups, with younger patients exhibiting a relatively higher risk (83). This finding highlights the need for increased attention to cardiovascular prevention in younger T2DM patients.

Cardiomyopathy is relatively uncommon in type 1 myotonic dystrophy (DM1) patients but remains an important complication. Patients with myotonic dystrophy may develop cardiomyopathy, primarily in the forms of dilated cardiomyopathy and hypertrophic cardiomyopathy (84). In contrast, the prevalence of myocardial dysfunction in type 2 diabetes mellitus (T2DM) patients can be as high as 75%. This dysfunction may be asymptomatic in the early stages but can persist for years, increasing the risk of progression to overt heart failure (85). Even in asymptomatic T2DM patients, myocardial perfusion reserve is significantly lower than that in healthy individuals (86). Diabetic cardiomyopathy is characterized by a high prevalence and insidious progression, while DM1-related cardiomyopathy is primarily defined by conduction abnormalities and structural heart disease. Both conditions necessitate long-term follow-up.

The occurrence of arrhythmias in type 1 myotonic dystrophy (DM1) is closely associated with ion channel dysfunction. Studies have found that mutations in the DMPK gene result in mis-splicing of the SCN5A gene, which encodes the cardiac sodium channel, leading to abnormal sodium current. This is a key mechanism underlying cardiac conduction block and arrhythmias (87, 88). Furthermore, animal model studies have confirmed that expanded CUG-repeat RNA reduces the activity of sodium and potassium channels, resulting in prolonged QRS and QTc intervals (89). Research indicates that dysregulation of microRNAs (miRNAs) may also play a role, with downregulation of miR-1 and upregulation of its target gene multiplexin (COL15A1) potentially promoting the development of dilated cardiomyopathy (90). Additionally, cell model studies have shown that DM1 cardiomyocytes exhibit mitochondrial dysfunction and energy metabolism defects, which may contribute to impaired myocardial function (32). Animal model research has also confirmed that the loss of MBNL protein function is an important factor in the cardiac contractile dysfunction and myocardial hypertrophy observed in DM1 (91). It is noteworthy that DM1 patients may also experience cardiac autonomic dysfunction. Although its precise role is still under investigation, it may exacerbate the occurrence of cardiac abnormalities and increase the risk of cardiovascular events (92).

### 3.5 Neurological system

The characteristic neurological impairments of type 1 myotonic dystrophy (DM1) include cognitive dysfunction, neuroendocrine disturbances, as well as personality and behavioral abnormalities (93). Most DM1 patients also exhibit frontal lobe dysfunction, with notable impairments in attention, executive functions, and visuospatial abilities (94, 95). Furthermore, a longitudinal study published in 2024 (96), which followed 38 DM1 patients for approximately five years, found significant declines in multiple cognitive domains, including the Mini-Mental State Examination, walking test, block design, and symbol-digit modality test. The “Chinese Expert Consensus on the Prevention and Treatment of Cognitive Dysfunction in Type 2 Diabetes Mellitus” (97) clearly states that individuals with type 2 diabetes have a significantly higher risk of developing cognitive dysfunction and dementia compared to the general population. The consensus emphasizes that type 2 diabetes can lead to impairments in multiple cognitive domains, primarily affecting language function, visuospatial abilities, executive function, motor skills, and information processing speed. Cognitive dysfunction may even onset during the prediabetic stage, often presenting insidiously, making diagnosis difficult and easily overlooked. Brain MRI scans show that long-term diabetic patients exhibit significant microvascular brain lesions, which are closely associated with cognitive impairment (98). Even in middle-aged patients with well-controlled diabetes and a relatively short duration of diagnosis (less than 10 years), hippocampal volume reduction and memory deficits may occur (99). Similarly, brain MRI scans of patients with type 1 myotonic dystrophy commonly reveal global gray matter reduction (predominantly in the frontal and parietal lobes), bilateral hippocampal volume reduction, and white matter lesions (100). These findings suggest that both type 1 myotonic dystrophy and type 2 diabetes may share similar structural brain changes when cognitive dysfunction develops.

### 3.6 Cancer risk

A large-scale study based on patient registries from Sweden and Denmark, involving 1,658 individuals with type 1 myotonic dystrophy, revealed that the overall cancer risk in these patients is twice as high as that of the general population (101). Special attention is needed for specific types of cancer in this patient group, including endometrial cancer, thyroid cancer, ovarian cancer, and skin melanoma (101, 102). Moreover, individuals with type 1 myotonic dystrophy also have an increased risk of developing thyroid nodules, colorectal polyps, uterine fibroids, as well as benign tumors in the brain and nervous system (103). A large-scale cohort study (104) found that the overall cancer incidence in individuals with type 2 diabetes is 16% higher than that in the general population. The study included 428,326 type 2 diabetes patients, with a total of 74,063 cancer cases identified during the follow-up period. Notably, the risks of liver cancer, pancreatic cancer, and colorectal cancer were significantly increased. Within the first 3 months following a diabetes diagnosis, the risks of colorectal, lung, liver, cervical, endometrial, ovarian, pancreatic, and prostate cancers were markedly elevated (105), showing a high degree of similarity with type 1 myotonic dystrophy. It is especially important to conduct timely cancer screening for newly diagnosed elderly patients with type 2 diabetes, with subsequent annual screenings (106). Notably, cancer risk in type 2 diabetes patients exhibits differences based on gender and age, with a significant increase in cancer risk across various sites in female patients, while male patients show no significant change in risk (107). This characteristic is also present in individuals with type 1 myotonic dystrophy (108).

### 3.7 Depression

Studies have shown that the weighted comorbidity prevalence of depression in individuals with type 1 myotonic dystrophy is 18% (109). Cognitive function and emotional regulation in these patients may be affected due to widespread white matter involvement (110). However, as individuals with type 1 myotonic dystrophy often present with multiple symptoms, these symptoms may interact with depression, with depression more likely to be a reactive adjustment disorder rather than a direct consequence of structural brain damage. According to the Chinese Guidelines for the Prevention and Treatment of Type 2 Diabetes (2020 Edition) (111), there exists a bidirectional relationship between type 2 diabetes and depression-anxiety disorders: on one hand, type 2 diabetes can promote the onset and progression of depressive and anxiety symptoms; on the other hand, depressive and anxious states can increase the risk of developing diabetes. Approximately 25% of diabetes patients experience varying degrees of depressive symptoms. Additionally, the guidelines highlight that the prevalence of depression and anxiety disorders is significantly higher in female diabetes patients compared to males. Pregnant and postpartum women with diabetes are at an elevated risk for developing depression and anxiety disorders. Furthermore, factors such as the age of diabetes onset, presence of complications, disease duration, glycemic control, and socioeconomic status are all associated with the occurrence of depression and anxiety. Papelbaum et al. (112) observed that patients with type 2 diabetes who also experience depressive symptoms have significantly higher levels of glycated hemoglobin compared to non-depressed patients. Glycated hemoglobin levels can serve as an important indicator of impaired mental health in individuals with type 2 diabetes.

The clinical management of type 1 myotonic dystrophy and type 2 diabetes requires a multidisciplinary approach that integrates comprehensive strategies. This includes regular screening for multi-system disease risks, as well as enhancing patient education to improve self-management skills. Emphasis should be placed on dietary control, appropriate physical activity, and mental health care. Through systematic monitoring and individualized treatment, overall prognosis can be improved.

## 4 Current therapeutic strategies

### 4.1 With a focus on blood glucose control at its core

Currently, there is a lack of curative treatments for type 1 myotonic dystrophy, and its survival rate is associated with factors such as age, diabetes, need for walking assistance, arrhythmias, blood pressure, and pulmonary function (113). Abnormal expression of the insulin receptor gene may play a role in the development of cancer in patients with type 1 myotonic dystrophy (114). As many cancer cell types also overexpress the IR-A isoform, this variant is believed to provide malignant cells with a selective growth advantage when exposed to insulin, thus increasing the risk of cancer and its progression (115). Therefore, strict blood glucose monitoring and management are of significant clinical importance for patients with type 1 myotonic dystrophy. Annual monitoring of fasting blood glucose and glycated hemoglobin is necessary, and 48 h continuous glucose monitoring can be used for early detection of abnormal glucose metabolism in these patients, with postprandial blood glucose levels serving as a key determinant (116). According to existing literature, when type 1 myotonic dystrophy coexists with type 2 diabetes, clinical treatment primarily focuses on blood glucose control. The following classes of antidiabetic medications are currently considered:

#### 4.1.1 Metformin

Metformin is a widely recognized first-line treatment for type 2 diabetes, effectively improving insulin resistance and blood glucose control. Research has confirmed (117) that metformin can correct splicing defects associated with DM1 by activating AMPK and downregulating the RNA-binding protein RBM3. In human embryonic stem cell derivatives and primary myoblasts from patients carrying DM1 pathogenic mutations, clinically used doses of metformin can induce this biological effect, providing direct molecular mechanistic support for metformin’s use in DM1 treatment. Furthermore, García-Puga et al. (32) discovered that fibroblasts and peripheral blood mononuclear cells from DM1 patients exhibit significant metabolic disturbances and mitochondrial dysfunction. This includes reduced oxidative phosphorylation activity, decreased ATP production, and increased accumulation of reactive oxygen species (ROS). Metformin treatment has been shown to reverse metabolic and mitochondrial defects in fibroblasts from DM1 patients and improve impaired cell proliferation, a hallmark of accelerated aging. This suggests that metformin may alleviate the pathological process of DM1 by enhancing cellular energy metabolism. On the other hand (118, 119), AMP-activated protein kinase (AMPK), as a key cellular energy sensor, is inhibited in DM1, and metformin, a known AMPK activator, is believed to exert its therapeutic effects by reactivating this pathway. By activating AMPK, metformin may regulate processes such as autophagy and apoptosis, thereby positively affecting RNA toxicity and muscle tissue and function. In DM1 patients, those with type 2 diabetes who did not receive metformin treatment exhibited a significantly higher cancer risk (hazard ratio [HR] = 3.60), while no such increased risk was observed in patients treated with metformin (HR = 0.43), indicating that metformin may provide a potential anticancer protective effect in DM1 patients by improving their metabolic status (8).

In addition to its metabolic benefits, the effect of metformin on the core symptom of DM1—muscle dysfunction—has also garnered significant attention, although the findings from various studies remain somewhat inconsistent. Bassez et al. (120) evaluated the efficacy of metformin (maximum tolerated dose of 3 grams per day, for 48 weeks) in 40 DM1 patients. Among the 23 patients who completed the study, the metformin-treated group (*n* = 9) showed an average improvement of 32.9 meters in the 6 min walk test, compared to just 3.7 meters in the placebo group (*n* = 14) (*P* < 0.05), indicating that metformin significantly improved patients’ physical capacity. The study also found that the improvement in physical performance was associated with an increase in total mechanical work, suggesting a positive effect on gait. However, the study also noted that the results did not reach significance in the intention-to-treat analysis, and no significant changes were observed in secondary endpoints such as muscle strength and myotonia, except for a modest reduction in body weight. Another observational cohort study presented at the 2025 CMR Global Conference (121) evaluated the effect of metformin on myocardial fibrosis and cardiac function in 43 boys with Duchenne muscular dystrophy (DMD), comparing them with a control group of 43 untreated subjects. The results revealed that, in patients with normal cardiac function, myocardial fibrosis significantly progressed in the control group, while no significant change was observed in the metformin group. This study suggests that early use of metformin may have a protective effect on the myocardium in DMD patients. Although the study cohort did not include DM1 patients, its findings provide indirect evidence for the potential benefits of metformin in dystrophy-associated cardiomyopathy. Despite the positive signals from the above study, the benefits of metformin were mainly observed in overall physical performance, with limited improvements in core muscle strength and myotonia. Additionally, metformin treatment is often associated with gastrointestinal adverse events, which, though generally mild to moderate, may affect patient tolerance and adherence (120). Therefore, the specific role and clinical value of metformin in improving muscle function require confirmation through larger-scale studies.

#### 4.1.2 Dipeptidyl peptidase-4 (DPP-4) inhibitors

Dipeptidyl peptidase-4 (DPP-4) inhibitors are a class of oral antidiabetic medications that promote insulin secretion and inhibit glucagon release in a glucose-dependent manner by prolonging the action of endogenous glucagon-like peptide-1 (GLP-1), thereby controlling blood glucose levels (122, 123). Due to their low risk of hypoglycemia and neutral effect on body weight, DPP-4 inhibitors are widely used in patients with type 2 diabetes (T2DM) (124, 125). A study published in 2024 (126) demonstrated that DPP-4 inhibitors showed potential benefits in patients with type 1 myotonic dystrophy (DM1), including a reduction in blood glucose levels in some patients, improvement of insulin secretion, and preservation of insulin secretory capacity. The study also revealed, through 48 h continuous glucose monitoring, that DM1 patients exhibit distinct glucose fluctuation patterns, suggesting that DPP-4 inhibitors may help improve these fluctuations. However, this study included only 29 patients with DM1 from Japan, which represents a small sample size, and did not provide data on the long-term safety and efficacy of DPP-4 inhibitors.

DPP-4 inhibitors have an overall favorable safety profile, with common adverse events including nasopharyngitis and headaches, and a low incidence of severe adverse events (127, 128). One of their most notable advantages is the very low risk of hypoglycemia, which is particularly important for patients with type 1 myotonic dystrophy (DM1), who may experience irregular eating patterns due to factors such as muscle weakness and swallowing difficulties (123, 124). Given that DM1 patients often have underlying cardiovascular issues, including cardiac conduction abnormalities, the cardiovascular safety of DPP-4 inhibitors has become a key clinical concern. Several large cardiovascular outcome trials have demonstrated that, compared to placebo, DPP-4 inhibitors do not increase the risk of major adverse cardiovascular events (MACE), indicating a neutral cardiovascular safety profile (129–131). However, a meta-analysis suggested that saxagliptin may be associated with an increased risk of heart failure-related hospitalizations (132), while another analysis found an increased risk of atrial fibrillation (RR = 1.52) associated with DPP-4 inhibitors (129). Given the elevated arrhythmia risk in DM1 patients, it is crucial to assess and monitor cardiac function when using DPP-4 inhibitors.

Furthermore, DPP-4 inhibitors may have potential effects on muscle function and fibrosis. A study in a heart failure mouse model (133) found that DPP-4 inhibitors, through the GLP-1 receptor signaling pathway, improved mitochondrial biogenesis in skeletal muscle, thereby enhancing exercise capacity. Another study (134) indicated that DPP-4 is a marker of activated fibroblasts, and in a model of systemic sclerosis (a fibrotic disease), DPP-4 inhibitors exhibited significant anti-fibrotic effects. Although these findings have not yet been validated in patients with type 1 myotonic dystrophy (DM1), they provide new avenues for exploring whether DPP-4 inhibitors could improve muscle function or slow the progression of fibrosis in DM1 patients.

However, the evidence for the use of DPP-4 inhibitors in patients with type 1 myotonic dystrophy (DM1) is primarily derived from observational studies or indirect inferences, with a lack of prospective randomized controlled trials (RCTs) specifically addressing their efficacy and safety in this population. While the cardiovascular safety of DPP-4 inhibitors has been established in the general type 2 diabetes mellitus (T2DM) population, the cardiovascular safety in DM1 patients, who already have a high risk of arrhythmias and heart failure, remains uncertain. In particular, the potential increased risk of atrial fibrillation and heart failure reported in some studies requires further investigation to determine whether these risks may be amplified in the DM1 population. Basic research suggests that DPP-4 inhibitors may have positive effects on muscle mitochondrial function and fibrosis (133, 134), but there is currently no evidence to support whether these effects translate into clinical benefits in terms of muscle strength, muscle stiffness, or disease progression in DM1 patients.

#### 4.1.3 Thiazolidinediones (TZDs)

Thiazolidinediones (TZDs), particularly pioglitazone, are insulin sensitizers that improve insulin sensitivity by activating peroxisome proliferator-activated receptor gamma (PPAR-*γ*), thereby modulating the expression of genes related to glucose and lipid metabolism. Several studies have confirmed that these agents can effectively improve insulin resistance and hyperinsulinemia in patients with type 1 diabetes mellitus (DM1). Yamamoto et al. (135) included eight DM1 patients with diabetes (mean age 52.1 years) in their study, administering a daily dose of 15 mg of pioglitazone, with an average follow-up duration of 14.8 months. The results demonstrated a significant improvement in the patients’ HOMA-IR score (a marker of insulin resistance), which decreased from 2.1 prior to treatment to 1.1 (*p* = 0.04). In the oral glucose tolerance test (OGTT), the insulin area under the curve (sigma IRI) over 120 min also showed a downward trend, particularly in patients with baseline hyperinsulinemia (sigma IRI ≥ 250 μU·hr./ml), where the decrease was more pronounced. A 2009 case study (136) reported two obese DM1 patients with poorly controlled blood glucose despite treatment with other antidiabetic medications or insulin (HbA1c levels of 11.8 and 10.3%, respectively). After 10 days of combined pioglitazone therapy, both patients experienced rapid improvement in their blood glucose levels, with one patient also showing a reduced insulin requirement. Additionally, a 2018 case report highlighted the improvement in blood glucose control in a DM1 patient with severe insulin resistance and hyperinsulinemia after using insulin sensitizers, including pioglitazone (137). These studies collectively suggest that pioglitazone effectively corrects the common hyperinsulinemia and insulin resistance in DM1 patients by enhancing peripheral tissue sensitivity to insulin. A 2024 retrospective case series (138) analyzed six DM1 patients presenting with diabetes as their initial symptom, revealing that the combined use of pioglitazone and metformin was effective in controlling blood glucose levels in these patients. These findings indicate that pioglitazone, whether used alone or in combination with other antidiabetic medications, can effectively improve blood glucose control in DM1 patients with concurrent diabetes, playing a key role in those with suboptimal control.

In the two obese DM1 patients with concurrent diabetes (136), after 10 days of pioglitazone treatment, not only was blood glucose improved, but levels of inflammatory markers in serum, including interleukin-6 (IL-6) and high-sensitivity C-reactive protein (hs-CRP), also decreased significantly, while the anti-inflammatory adiponectin levels notably increased. This suggests that the therapeutic effects of pioglitazone may, in part, be attributed to its anti-inflammatory action. However, whether this mechanism is universally applicable to a broader DM1 patient population, and its potential association with improvements in muscle function, remains to be further explored. Despite these promising findings, most existing studies on pioglitazone treatment for DM1 are small case reports, case series, or retrospective studies, lacking large-scale, prospective randomized controlled trials (RCTs) to definitively confirm its efficacy and safety. The long-term effects of pioglitazone in DM1 patients, including its potential impact on cardiovascular events and fractures, are still unclear. Although pioglitazone has been observed to improve insulin resistance and reduce inflammatory markers, whether it acts by directly correcting the core pathological mechanisms of DM1 (e.g., correcting splicing abnormalities) or through other signaling pathways (such as the AKT pathway) requires further in-depth molecular research to clarify (119).

#### 4.1.4 GLP-1 receptor agonists

Glucagon-like peptide-1 receptor agonists (GLP-1R agonists) are a class of drugs originally developed for the treatment of type 2 diabetes (119). Their mechanism of action involves mimicking the endogenous incretin GLP-1, which stimulates insulin secretion, inhibits glucagon release, delays gastric emptying, and acts on the central nervous system to enhance satiety. These combined effects contribute to blood glucose control and weight reduction (119, 139). As research progresses, the pleiotropic effects of GLP-1R agonists have become increasingly apparent, with growing interest in their potential benefits in cardiovascular protection, neuroprotection, anti-inflammation, and metabolic regulation (119, 140, 141). GLP-1 receptors are widely distributed in various tissues and organs, including the pancreas, heart, brain, and muscles, providing a theoretical foundation for their application in multiple disease areas (119). Current evidence suggests that GLP-1R agonists may offer an effective therapeutic option for patients with DM1.

A case report published in 2024 described a 47-year-old female patient with type 1 diabetes mellitus (DM1) and poorly controlled blood glucose levels. After standard treatment with pioglitazone and insulin proved ineffective, the addition of a GLP-1R agonist led to significant improvement in the patient’s blood glucose levels, ultimately allowing for the successful discontinuation of insulin. Genetic testing revealed that the patient carried a heterozygous mutation (p.R131Q, rs3765467) in the GLP-1 receptor (GLP1R) gene, a variant known to be associated with increased endogenous insulin secretion following exogenous GLP-1 infusion. This report is the first to highlight how GLP-1R gene polymorphisms may influence the therapeutic response to GLP-1R agonists in DM1 patients, suggesting that these drugs may exert a more pronounced hypoglycemic effect in DM1 patients with specific genetic backgrounds (142). Although large-scale clinical trials directly evaluating the impact of GLP-1R agonists on muscle function in DM1 patients are currently lacking, some basic research and reviews on other myopathies provide theoretical support for this direction. A review published in 2023 explored the potential application of GLP-1R agonists in idiopathic inflammatory myopathies (IIM). This systematic review of 19 studies found that GLP-1R agonists could alleviate muscle atrophy, inflammation, and weakness to varying degrees in both mouse and human studies. Additionally, they improved muscle microvascular structure, endurance, and promoted mitochondrial biogenesis. These findings suggest that GLP-1R agonists have the potential to improve muscle function and structure, emphasizing the need for randomized controlled clinical trials in patients with myopathies such as IIM (143). While the review was not specifically focused on DM1, the mechanisms revealed for the protective effects of GLP-1R agonists on muscle provide valuable insights for exploring their application in the treatment of DM1. Although GLP-1R agonists hold potential benefits for DM1 patients, evidence regarding their effectiveness and safety remains insufficient, and their direct impact on muscle function and disease progression is yet to be determined.

#### 4.1.5 Pentamidine (PTM)

Pentamidine (PTM) is a drug initially developed as a synthetic analog of insulin, which has since received joint approval from the U.S. Food and Drug Administration (FDA) and the European Medicines Agency (EMA) for the clinical treatment of various parasitic infections (144, 145). PTM is the first small molecule proposed for the treatment of type 1 myotonic dystrophy (DM1), as it has been shown to reverse the splicing defects associated with this condition. Consequently, PTM has been suggested as a novel potential therapeutic candidate for the treatment of psychiatric disorders, myotonic dystrophy, diabetes, and cancers (146).

Several basic research studies have confirmed that pentamidine directly targets the core pathological mechanisms of DM1. Warf et al. (147) were the first to demonstrate that pentamidine can interfere with the binding of MBNL1 protein to CUG repeat sequences *in vitro*. In their study, the researchers identified pentamidine and neomycin B as inhibitors of this interaction; however, only pentamidine was found to effectively reverse the mis-splicing of two pre-mRNAs affected by DM1 in a cell culture model. Furthermore, pentamidine significantly reduced the formation of RNA aggregates in the nucleus and released sequestered MBNL1 protein. In addition, in a mouse model expressing amplified CUG repeat sequences, pentamidine partially restored the splicing defects. Another study further validated the therapeutic potential of pentamidine in a DM1 Drosophila model, which specifically expressed toxic CUG repeat sequences in cardiomyocytes to mimic the cardiac dysfunction associated with DM1. The researchers found that feeding pentamidine to the flies not only reduced RNA aggregates in the heart but also improved arrhythmia and contractile function, significantly increasing the survival rate of the flies (148). These studies collectively suggest that pentamidine improves the molecular and functional manifestations of DM1 by releasing MBNL proteins and reducing RNA toxicity.

Despite its therapeutic potential demonstrated in preclinical models, the clinical translation of pentamidine faces significant challenges, primarily due to its inherent toxicity. Chakraborty et al. (149) noted that pentamidine exhibits cytotoxicity at high concentrations. Moreover, the nephrotoxicity of pentamidine limits its systemic administration in DM1 treatment (150). To overcome these obstacles, researchers have explored various strategies. One approach involves identifying and developing pentamidine analogs with lower toxicity. Chakraborty et al. (149) evaluated a series of pentamidine derivatives and discovered that one compound, furamidine (compound 13), was equally effective in reversing splicing errors as pentamidine but exhibited significantly reduced toxicity. Another study identified a novel pentamidine analog, EBAB, which showed markedly lower *in vivo* toxicity compared to pentamidine (151). Moreover, a 2019 study found that combining erythromycin with the prodrug of furamidine (pafuramidine) exhibited a synergistic effect in correcting splicing errors in a DM1 mouse model and partially alleviated the myotonic phenotype (152). Another strategy to address the toxicity of pentamidine is to improve the drug delivery system to achieve targeted delivery and reduce systemic toxicity. Repellin et al. (153) proposed using hyaluronic acid-based nanoparticles as carriers to encapsulate pentamidine. *In vitro* studies showed that these nanoparticles were efficiently internalized by skeletal muscle cells, demonstrated good safety profiles, and effectively reduced RNA aggregation in the nuclei of DM1 cell models. Similarly, mesoporous silica nanoparticles (MSNs) have been explored as carriers for pentamidine. Functionalized MSNs (MSN-COOH) were able to efficiently load pentamidine and achieve controlled drug release, providing a potential approach to reduce its toxicity (150). These studies suggest that through chemical structural modifications or innovative drug delivery systems, it may be possible to overcome the toxicity barriers of pentamidine and facilitate its clinical translation.

It is particularly important to note that both type 1 myotonic dystrophy (DM1) and type 2 diabetes are metabolic disorders associated with high cardiovascular risk. The 2019 guidelines on dyslipidemia management from the European Society of Cardiology and the European Atherosclerosis Society set stricter treatment targets for lipid-lowering therapy. For patients at high cardiovascular risk, they recommend reducing low-density lipoprotein cholesterol (LDL-C) levels by 50% compared to baseline, with a target level of <1.8 mmol/L (<70 mg/dL). Therefore, strict lipid control is of significant clinical importance in the comprehensive management of patients with DM1 and type 2 diabetes. However, the increased degree of frailty in DM1 is associated with hypothyroidism and the use of certain cholesterol-lowering drugs, particularly statins. A combination of ezetimibe and bempedoic acid has been shown to be effective for patients intolerant to statins and those at high cardiovascular risk. Bempedoic acid offers a viable option for reducing LDL-C without causing significant muscle-related side effects (154) (see Table 1).

**Table 1:** The effects, mechanisms of action, current research status, and future research directions of various antidiabetic drugs for type 1 myotonic dystrophy complicated with type 2 diabetes are outlined above.

Drug class	Pharmacological Properties	Mechanism of action	Support from evidence	Current state of research	Future directions
Metformin	It is administered orally and absorbed in the small intestine, typically formulated as an emulsion or enteric-coated tablet.	1. Activation of AMPK; 2. Inhibition of RBM3 RNA-binding protein; 3. Correction of DM1-associated splicing defects; 4. Improvement of insulin resistance and blood glucose control.	Numerous clinical studies, including both animal models and clinical trials, support this.	It demonstrates superior efficacy compared to traditional antidiabetic medications, with a mechanism that directly targets the pancreas, and shows promising results in various models.	1. Conduct large-scale Phase III clinical trials to evaluate the long-term efficacy and safety of DM1 patients, focusing on physical function, quality of life, and muscle function. 2. Explore biomarker-guided individualized treatment approaches, incorporating molecular and imaging biomarkers for treatment monitoring. 3. Conduct head-to-head comparison studies and combination therapy trials to investigate the synergistic effects with antispasmodic drugs and RNA-targeted therapies. 4. Focus on the preventive role in complications, assessing the long-term effects of metformin in preventing DM1-related complications.
DPP-4 inhibitors	Oral administration, rapid absorption, and prolonged duration of action.	1. Inhibition of DPP-4 enzyme activity, thereby prolonging the half-life of GLP-1; 2. Promotion of insulin secretion and reduction of blood glucose levels.	Clinical studies have demonstrated favorable glucose-lowering effects and improvements in insulin secretion.	The treatment demonstrates good glycemic control in patients with DM1, particularly when used in combination therapy.	1. Conduct high-quality randomized controlled trials to evaluate the efficacy and safety of DPP-4 inhibitors in DM1 patients with concomitant metabolic abnormalities. 2. Enhance cardiovascular safety monitoring by assessing the incidence of cardiovascular events such as arrhythmias and heart failure. 3. Explore the potential impact on muscle function by evaluating muscle function indicators, such as grip strength and walking tests. 4. Perform subgroup analyses and biomarker studies to identify treatment responses and safety across different patient subgroups.
Pioglitazone	Oral administration, or oral or injectable routes.	1. Improvement of insulin resistance; 2. Reduction of inflammatory markers; 3. Indirect improvement of glucose control; 4. However, the underlying mechanisms remain unclear, and its direct effect on the core pathological mechanisms of type 1 diabetes (DM1) has not been definitively demonstrated.	Preclinical studies provide supporting evidence; however, the clinical trials have small sample sizes, and the long-term efficacy and safety remain uncertain.	This approach has not yet been widely implemented in the treatment of DM1, and further studies with larger sample sizes are required to validate its efficacy.	1. Conduct high-quality clinical trials to evaluate the efficacy and safety of treatment in patients with DM1. 2. Perform long-term follow-up studies to assess the sustained effectiveness of therapy and monitor adverse events such as heart failure, edema, and fractures. 3. Explore the application in specific subgroups by assessing whether pioglitazone can delay the onset of diabetes or improve muscle function in DM1 patients without diabetes but with insulin resistance. 4. Strengthen basic and translational research to investigate the mechanisms of pioglitazone in DM1, including its effects on DMPK gene expression and RNA toxicity.
GLP-1 receptor agonists	It can be administered orally or via injection.	1. Activation of GLP-1 receptors; 2. Promotion of insulin secretion; 3. Inhibition of glucagon release; 4. Anti-inflammatory effects; 5. Blood glucose control associated with insulin resistance.	Multiple basic studies support this, including in vitro and in vivo research as well as mouse models.	Genetic testing may be required to determine the appropriate indications, as significant differences in drug response exist due to variations in genetic backgrounds.	1. Conduct prospective clinical studies to evaluate the efficacy and safety of different GLP-1 receptor agonists in patients with DM1. 2. Perform pharmacogenetic studies to explore the relationship between GLP-1R gene variations and drug efficacy. 3. Investigate the direct effects on muscle pathology by studying whether GLP-1 receptor agonists can improve muscle inflammation, fibrosis, and other pathological changes. 4. Design studies targeting specific subgroups to assess the potential value of GLP-1 receptor agonists in DM1 patients with concomitant metabolic syndrome.
Pentamidine (PTM)	Orally administrable, it can serve as a glucose loading factor or a carrier drug.	1. Reversal of splicing defects; 2. Reduction of RNA toxicity; 3. Improvement of cardiac function; 4. Decrease in arrhythmias and contractile dysfunction.	Multiple in vivo and in vitro studies support this, including those conducted in mouse models and expression clone models.	Key strategies to overcome toxicity issues:1. Identify analogs with lower toxicity, such as furamidine and EBAB. 2. Develop innovative drug delivery systems, such as hyaluronic acid nanoparticles.	1. Clinical translational feasibility remains unknown: There is a lack of clinical trial evidence in humans, and the safety and efficacy have not been clearly defined. 2. Balancing toxicity and therapeutic window: Assess the impact of cytotoxicity and nephrotoxicity on clinical application. 3. Precise details of the mechanism of action: Further investigation is needed into the molecular targets and mechanisms, particularly the mechanism of binding with CUG-MBNL1.

### 4.2 Emerging therapies targeting the core pathogenesis of DM1

#### 4.2.1 Antisense oligonucleotides

The core pathogenic mechanism of DM1 involves the production of toxic RNA, making direct targeting of the mutant DMPK RNA transcript an attractive therapeutic strategy (155). Antisense oligonucleotides (ASOs) are synthetically designed short-chain nucleic acid molecules that can bind to specific mRNA sequences through base-pairing principles, thereby modulating gene expression (156). Several studies have demonstrated that ASOs effectively reduce the levels of mutant DMPK transcripts, decrease the formation of RNA foci in the cell nucleus, and promote the release and nuclear redistribution of sequestered MBNL proteins (157). With ongoing advancements in ASO chemical modifications and delivery technologies, ASO-based therapies have shown tremendous potential in the treatment of various neuromuscular diseases, including DM1, and have become a focal point of current research (158).

The core mechanism of ASO-based therapy for DM1 lies in targeting and eliminating or neutralizing the toxic CUG-expansion RNA produced by the DMPK gene mutation. Research in this area primarily follows two technical approaches: one involves using RNase H-dependent ASOs (gapmers) to degrade the toxic RNA transcripts, and the other employs steric-blocking ASOs to prevent the binding of toxic RNA to proteins such as MBNL1, thereby releasing these proteins and restoring their normal splicing regulatory function. Several preclinical studies have confirmed the effectiveness of these strategies. Lee et al. (159) successfully induced RNase H-mediated degradation of CUG repeat transcripts in DM1 cell cultures and animal models using chimeric ASOs (gapmers), effectively disrupting RNA foci. Another study demonstrated that systemic application of a 2′-4′-constrained ethyl-modified ASO (ISIS 486178) in the DMSXL mouse model resulted in a 70% reduction of CUG-expanded RNA in skeletal muscle and a 30% reduction in the heart, significantly improving the mice’s body weight, muscle strength, and histological features of muscle tissue (160). A 2020 study further confirmed that ASOs targeting non-CUG sequences within the 3’ UTR of DMPK (ISIS 486178) similarly reduced RNA foci and DMPK mRNA levels in the heart and skeletal muscle, improved splicing defects, and reversed key DM1 phenotypes, including muscle stiffness and cardiac conduction abnormalities (161).

In terms of the blocking strategy, studies have shown that short locked nucleic acids (all-LNAs) complementary to CUG repeats can effectively reduce RNA foci in DM1 cell cultures and animal models, and efficiently and specifically correct MBNL1-sensitive splicing defects (162). Comparison between the two strategies revealed that both gapmers and repeat sequence blockers can reduce DMPK transcript levels and decrease RNA foci. However, repeat sequence blockers demonstrated higher efficiency in restoring MBNL1 function and correcting splicing defects, with fewer off-target effects at the transcriptome level (163). Collectively, these studies provide a solid theoretical and experimental foundation for ASOs as a potential therapeutic approach for DM1.

Despite significant preclinical advancements, early ASO therapies face substantial challenges in clinical application. These challenges primarily stem from the vulnerability of unmodified ASOs to nucleases *in vivo*, as well as their hydrophilic and negatively charged properties, which hinder their ability to cross cell membranes. As a result, their bioavailability in key target tissues, particularly skeletal muscle and the heart, remains low (155). A clinical trial assessing the safety of the ASO drug baliforsen in DM1 patients reported overall good tolerance. However, the drug’s concentration in skeletal muscle was lower than expected and did not achieve significant reductions in the target gene levels, highlighting the urgent need for improved muscle delivery (164). To address these challenges, researchers have developed various chemical modifications and novel delivery systems. Chemical modifications, such as 2’-O-methyl and 2′, 4′-constrained ethyl modifications, can enhance the stability of ASOs and their affinity for target RNAs (165). More importantly, conjugating ASOs with specific ligands—such as cell-penetrating peptides (CPPs), fatty acids, or antibodies targeting specific cell surface receptors like transferrin receptor 1 (TfR1)—has become a mainstream strategy to improve targeted delivery efficiency (157).

Several studies have demonstrated the potential of these delivery systems. One study showed that a PMO conjugated with the arginine-rich cell-penetrating peptide Pip6a (Pip6a-PMO-CAG) effectively reversed splicing defects and myotonia in DM1 mice at low doses, achieving long-term phenotypic correction (165). Additionally, ASOs conjugated with the C16 fatty acid ligand (IONIS-877864) were found to reduce mutant hDMPK transcripts in the skeletal muscles of DMSXL mice by 92% compared to unmodified ASOs, significantly improving muscle strength (166). Recent conference abstracts have also reported the development of novel drugs, such as antibody-oligonucleotide conjugates (e.g., del-desiran) and oligonucleotides with enhanced delivery (e.g., PGN-EDODM1), which have shown good tissue targeting and preliminary efficacy in preclinical models and early clinical trials (167). For example, a Phase 3 clinical trial design report for del-desiran indicated that the drug aims to deliver siRNA to muscle cells by targeting TfR1 (168). Similarly, non-clinical data for PGN-EDODM1 demonstrated efficient nuclear delivery using cell-penetrating peptide technology, which almost completely resolved the pathological features of DM1 in HSALR mice (169). These innovative strategies pave the way for the successful application of ASO therapies in DM1.

#### 4.2.2 AAV-based gene therapy

Several preclinical studies have demonstrated that using AAV vectors to deliver therapeutic nucleic acid molecules can effectively intervene in the core pathological mechanisms of DM1, reversing disease phenotypes in both cellular and animal models.

A study presented at the 2024 WMS Annual Meeting (170) designed an improved U7 small nuclear RNA (U7snRNA), delivered via an AAV vector, aimed at promoting the downregulation of the DMPK gene or inhibiting the effect of CUG repeat sequences. In cell lines derived from DM1 patients, AAV treatment successfully reduced the number of nuclear foci, released sequestered MBNL1 protein, and improved the RNA splicing profile. In subsequent animal experiments, researchers injected AAV1.U7snRNAs into HSAlr mouse models, which express CUG repeats exclusively in skeletal muscle. After eight weeks of intervention, functional tests showed a significant reduction in muscle stiffness, with some muscles no longer exhibiting electrical myotonia. Molecular-level analysis further confirmed that following treatment, the number and intensity of nuclear foci in muscle tissue were reduced, along with the correction of splicing defects in several key genes, including Serca1, Mbnl1, Ldb3, and Clcn1. A similar study presented at the 2024 Annual Meeting of the American Society of Gene and Cell Therapy (171) reached comparable conclusions. In this study, researchers also used AAV to deliver U7snRNA and observed a significant reduction in myotonia and improvements in Serca1 and Clcn1 splicing in the DM1 mouse model, further validating the effectiveness of this strategy both *in vitro* and *in vivo*.

In addition to U7snRNA, CRISPR technology has also been integrated into the AAV delivery system for exploring treatments for DM1. The AAV vector delivers the CRISPR Cas13b system, which specifically targets and cleaves the mRNA transcript of DMPK (170). In preliminary experiments conducted on immortalized myoblasts derived from DM1 patients (172), the results showed that Cas13-mediated DMPK knockdown effectively reduced MBNL nuclear foci and corrected splicing defects. The research team now plans to conduct further in vivo experiments in mouse models to validate its efficacy.

These studies collectively demonstrate that AAV-based gene therapy strategies, whether through U7snRNA to interfere with CUG repeat sequences or through the CRISPR Cas13 system to degrade DMPK mRNA, can effectively improve the molecular and functional characteristics of DM1. These findings provide a solid scientific foundation for the development of AAV-based gene therapies for human DM1 (170–172).

#### 4.2.3 CRISPR/Cas9

In recent years, gene editing technologies, exemplified by CRISPR/Cas9, have opened new possibilities for fundamentally correcting the genetic defects underlying DM1 (173). The CRISPR/Cas9 system allows precise targeting and cleavage of specific genomic loci, providing a powerful tool for the permanent elimination of pathogenic CTG repeats or suppression of their transcription. This approach holds the potential to reverse the molecular pathological processes of the disease at its source (174).

##### 4.2.3.1 Permanent correction of genetic defects through the removal of CTG repeats

The most direct and widely studied strategy for DM1 treatment using CRISPR/Cas9 technology is the permanent removal of the pathogenic CTG repeat expansion from the genome. Studies have shown (175) that the CRISPR/Cas9 system, through dual cuts flanking the CTG repeat region, successfully removed the toxic repeat sequence in myogenic cells derived from fibroblasts of DM1 patients. Following gene editing, typical disease markers, such as RNA aggregates containing CUG repeats, disappeared, and the abnormal splicing patterns of downstream genes were restored, thereby allowing the affected cells to revert to a normal phenotype. Dastidar et al. (176) also validated the efficacy of this strategy in induced pluripotent stem cells (iPSCs) derived from DM1 patients and their differentiated muscle cells. Using a dual-guide RNA (gRNA) approach, they achieved up to 90% efficiency in CTG repeat removal, observing the disappearance of RNA aggregates, restoration of normal MBNL1 protein localization, and normalization of SERCA1 splicing patterns.

At the animal model level, several studies have further confirmed the *in vivo* efficacy of this strategy. One study used the *Staphylococcus aureus* Cas9 (SaCas9) system and delivered the CRISPR components via a single injection of an AAV vector into the tibialis anterior muscle of the DMSXL mouse model (which carries over 1,000 CTG repeats of the human DMPK gene). This approach successfully removed the CTG repeat sequence and significantly reduced the number of pathological RNA aggregates in muscle cell nuclei (177). Another study similarly delivered the CRISPR/Cas9 components systemically via AAV in a DM1 mouse model, achieving excision of the CTG expansion in both cardiac and skeletal muscles. This intervention not only significantly improved molecular defects but also led to observable phenotypic improvements, such as increased body weight, enhanced muscle strength, and improved body composition in the mice (178). These studies collectively demonstrate that CRISPR/Cas9-mediated removal of the CTG repeat sequence is a feasible therapeutic strategy capable of permanently correcting the genetic defect and reversing the core pathological features of DM1 (157).

##### 4.2.3.2 Intervention through suppression of mutant allele transcription

In addition to directly excising the repeat sequence, another strategy involves using CRISPR technology to suppress the transcription of the mutant DMPK allele, thereby reducing the production of toxic CUG repeat RNA at its source. This approach is primarily achieved through CRISPR interference (CRISPRi) or by inserting a termination signal upstream of the repeat sequence. Wang et al. (158) explored targeting the 3’ UTR upstream of the CTG repeat sequence to insert a polyadenylation (polyA) signal. The results showed that this method effectively terminated transcription of the mutant allele, thereby eliminating the production of toxic RNA. This approach successfully reversed disease-related phenotypes in differentiated neural stem cells, neurons, cardiomyocytes, and skeletal muscle fibers. The CRISPRi technique utilizes a catalytically inactive Cas9 (dCas9) protein, which retains its ability to bind to target DNA but loses its cutting activity. When dCas9 is paired with a specific gRNA to target the promoter region of the DMPK gene, it effectively blocks RNA polymerase binding or elongation, thereby inhibiting gene transcription. Porquet et al. (179) systematically evaluated different sgRNAs targeting the DMPK promoter and found that the most effective sgRNA reduced the levels of both DMPK transcripts and CUG-expanded RNA aggregates by up to 80%. This suppression specifically corrected splicing abnormalities across the global transcriptome and reversed the physiological defects in DM1 muscle cells. Whole-genome expression analysis confirmed that the effects were limited to the DMPK gene. Furthermore, dCas9 can be used to directly target the CTG repeat sequence, blocking the transcriptional process of RNA polymerase II through steric hindrance (173). These studies suggest that transcriptional suppression strategies provide an alternative therapeutic approach for DM1, one that does not require DNA cleavage, potentially reducing the risks of off-target effects and genomic rearrangements (180).

##### 4.2.3.3 Direct targeting of toxic RNA degradation

Unlike interventions at the DNA level, the CRISPR system can also be engineered to directly target and degrade the toxic CUG repeat RNA generated within the cell nucleus. Researchers utilized a catalytically inactive Cas9 (dCas9) combined with a gRNA targeting the CUG repeat sequence, delivered via AAV vectors for muscle or systemic injection in a DM1 mouse model. The results demonstrated that dCas9 expression could be sustained for up to three months and successfully achieved multiple therapeutic outcomes: it eliminated toxic RNA aggregates in the nucleus, restored the normal distribution of the splicing factor MBNL1, reversed the abnormalities of various splicing biomarkers, and ultimately improved the muscle stiffness symptoms in the mice (181). Another study proposed a strategy using dCas9 fused with ribonuclease (RNase) to directly degrade the expanded CUG RNA (174). This RNA-level intervention strategy avoids permanent alterations to the genomic DNA, offering a safe and effective alternative for treatment.

Despite the promising results of CRISPR/Cas9-based DM1 therapies in preclinical studies, several challenges remain for their clinical application. First, delivery efficiency and specificity are critical bottlenecks. There is a need to develop efficient delivery systems capable of specifically targeting the affected tissues, such as skeletal muscle and the heart. While AAV vectors are the most widely studied tool, their vector capacity, immunogenicity, and transduction efficiency in different tissues still require optimization (173). Second, safety concerns are paramount. The CRISPR/Cas9 system may induce unintended off-target cuts, causing DNA double-strand breaks at other similar genomic loci, which could potentially lead to carcinogenic risks or functional gene disruption (173). Furthermore, even if cutting occurs at the intended target, unexpected genomic rearrangements, such as large inversions or deletions, may also arise (182). A study using a dual-cutting strategy to excise the CTG repeat sequence unexpectedly revealed substantial off-target effects and targeted genomic rearrangements, highlighting the need for comprehensive evaluation of editing outcomes and consideration of alternative approaches, such as CRISPRi, which do not rely on DNA double-strand breaks (180). Finally, the host’s immune response to the Cas9 protein and AAV vectors is another critical issue. Pre-existing or treatment-induced antibodies against the Cas9 protein or AAV capsid could impact therapeutic efficacy and trigger immune-related adverse events (173). Therefore, addressing these challenges related to delivery, safety, and immunogenicity must be systematically resolved before advancing to clinical trials (183).

#### 4.2.4 Other RNA-targeting therapeutic strategies

In addition to direct degradation or inhibition of DMPK RNA using antisense oligonucleotides (ASOs), researchers are also exploring other indirect strategies to modulate RNA toxicity.

**Figure 2:**
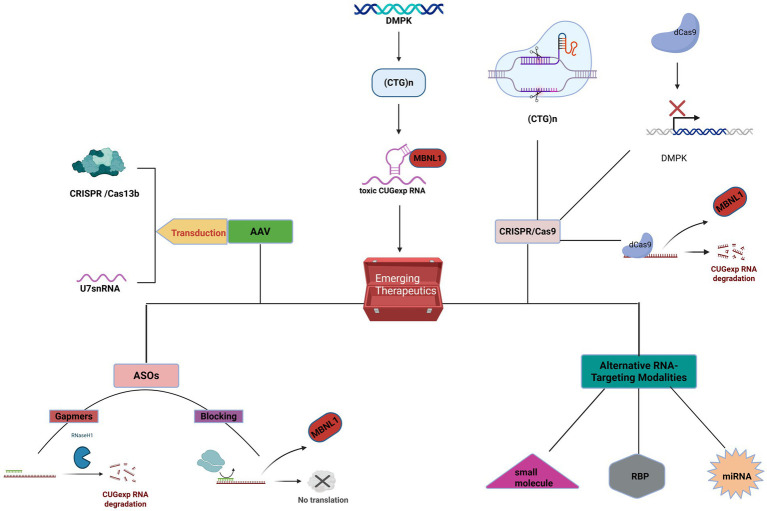
The general mechanisms of RNA-targeted therapies aimed at the core pathogenic mechanisms of DM1, including antisense oligonucleotides, AAV-based gene therapy, CRISPR/Cas9, and other RNA-targeted treatments, are illustrated as shown above.Figure created in BioRender. Luo, L. (2025) https://BioRender.com/nmxu50v.

1. Targeting MicroRNAs (miRNAs): Studies have shown that in DM1, miRNAs such as miR-23b and miR-218 suppress the translation of the MBNL1 protein (184). Therefore, developing antisense oligonucleotides (ASOs) targeting these miRNAs (antimiRs) has emerged as a promising strategy. Several studies have demonstrated that using antimiRs conjugated with cell-penetrating peptides effectively increases endogenous MBNL1 protein levels, improving splicing defects and muscle function phenotypes in DM1 cell and mouse models (184). Anti-miR treatment has proven effective in primary myoblasts from patients with varying CTG repeat lengths and, in addition to enhancing MBNL1 protein synthesis, unexpectedly reduced DMPK transcripts and RNA aggregates, suggesting a dual mechanism of action (185).
2. Small Molecule Drugs: Small molecule drugs have garnered significant attention due to their advantages in oral bioavailability and ability to penetrate biological barriers. Through screening, Gibaut et al. (186) identified a small molecule that specifically binds to r(CUG)exp. and, based on this finding, developed a chimeric degrader. This degrader selectively degrades transcripts containing the repeat sequence in DM1 myotube cells, significantly improving DM1-associated splicing defects with limited off-target effects.
3. Engineered RNA-Binding Proteins: Arandel et al. (187) designed an engineered RNA-binding protein (RBP) to act as a “decoy” for CUGexp. In DM1 cell and mouse models, delivery of this decoy protein via AAV vectors effectively binds to CUGexp, thereby releasing sequestered endogenous MBNL1. This approach long-term corrects splicing defects and improves disease pathology (see Figure 2).

## 5 Discussion

In summary, there is a complex association between type 1 myotonic dystrophy (DM1) and type 2 diabetes in terms of epidemiology, pathogenesis, and clinical management. The incidence of type 2 diabetes in DM1 patients is significantly higher than in the general population, which may be related to the progressive worsening of insulin resistance and *β*-cell dysfunction. Additionally, the onset of diabetes occurs earlier in DM1 patients compared to typical type 2 diabetes patients, suggesting a synergistic effect of genetic background and metabolic dysregulation. Furthermore, the severity of DM1 is positively correlated with diabetes risk, especially in late-onset patients, who are more likely to develop metabolic complications. This highlights the need for clinicians to conduct long-term blood glucose monitoring in DM1 patients. The pathophysiological process of DM1 complicated by type 2 diabetes involves multiple factors. On one hand, DMPK gene mutations lead to RNA toxicity, causing abnormalities in insulin signaling pathways in skeletal muscle and adipose tissue, thus triggering insulin resistance. On the other hand, muscle atrophy and limited mobility associated with DM1 exacerbate metabolic dysfunction, creating a vicious cycle. Additionally, some patients may exhibit concurrent pancreatic *β*-cell dysfunction, which aligns with the systemic nature of type 1 myotonic dystrophy (DM1) involving visceral organs. Although several mechanisms, including RNA toxicity, post-receptor signaling defects, loss of DMPK protein function, and inflammation, have been implicated in insulin resistance in DM1, the relative importance and interactions of these mechanisms across different patients and disease stages remain unclear. Current research has predominantly focused on skeletal muscle, yet DM1 is a multisystemic disorder. The specific molecular mechanisms and pathophysiological roles of the liver and adipose tissue in DM1-related insulin resistance require further investigation. Additionally, the precise connection between insulin resistance and the hallmark symptoms of DM1, such as muscle atrophy and weakness, remains to be fully elucidated.

Patients with type 1 myotonic dystrophy (DM1) complicated by type 2 diabetes exhibit a higher risk of multisystemic complications. Since DM1 itself can lead to cardiac conduction abnormalities and respiratory dysfunction, hyperglycemia may accelerate the progression of these comorbidities. Currently, there is no specific therapy for DM1 complicated by type 2 diabetes; however, comprehensive management can significantly improve patients’ quality of life. Lifestyle interventions form the foundation of treatment, including nutritional support and moderate rehabilitation exercises to mitigate muscle atrophy. Existing antidiabetic medications show potential in certain areas, but their long-term safety and efficacy remain insufficiently documented. Further optimization, development, and evaluation of the most effective glucose-lowering drugs are necessary. Emerging targeted therapies, such as antisense oligonucleotides, have demonstrated significant improvements at the molecular level. However, large-scale clinical trials are yet to be completed, and the long-term safety, tolerability, and clinical translation potential require further validation.

## 6 Conclusion

In recent years, significant progress has been made in understanding the mechanisms linking type 1 myotonic dystrophy (DM1) with type 2 diabetes. As a rare neuromuscular disease model, DM1 provides a unique perspective for deeply understanding the role of skeletal muscle in insulin resistance, while also contributing to the broader knowledge of the pathogenesis of type 2 diabetes. However, further research is needed to elucidate the complete molecular pathway from genetic mutations to clinical manifestations, and to better explore insulin signaling abnormalities in DM1 patients, particularly the impact of insulin receptor splice variants on insulin sensitivity and secretion. Currently, large-scale, multicenter, longitudinal studies assessing the long-term health outcomes of DM1 patients with type 2 diabetes are lacking. Such studies are crucial for documenting the onset, progression, and related complications of type 2 diabetes in this population, improving our understanding of its natural history in DM1, evaluating the long-term efficacy and safety of existing glucose-lowering agents, and developing targeted therapies specifically addressing insulin resistance in DM1. Furthermore, optimizing metabolic management strategies for DM1 patients is essential to enhance their quality of life and reduce multisystem complications. Through interdisciplinary collaboration, future research is expected to provide deeper insights into the common pathological mechanisms and individualized treatment strategies for these two diseases, ultimately improving clinical outcomes for patients. Specifically, RNA-targeted therapies for type 1 myotonic dystrophy (such as antisense oligonucleotides) and muscle repair therapies may represent key breakthrough areas. However, research on DM1 combined with type 2 diabetes remains limited, with current studies primarily focused on skeletal muscle tissue, while the pathological mechanisms in the liver and other internal organs are not yet well understood. Furthermore, there is a lack of comprehensive understanding of the complexity of the underlying mechanisms, and individual differences among patients, as well as disease progression, pose additional challenges in elucidating shared mechanisms. Therefore, integrating current research and further exploring multi-targeted therapeutic strategies remains a critical challenge.
